# On the stiffness of surfaces with non-Gaussian height distribution

**DOI:** 10.1038/s41598-021-81259-8

**Published:** 2021-01-21

**Authors:** Francesc Pérez-Ràfols, Andreas Almqvist

**Affiliations:** Division of Machine Elements, Luleå University of Technology, 97187 Luleå, Sweden

**Keywords:** Mechanical engineering, Applied physics

## Abstract

In this work, the stiffness, i.e., the derivative of the load-separation curve, is studied for self-affine fractal surfaces with non-Gaussian height distribution. In particular, the heights of the surfaces are assumed to follow a Weibull distribution. We find that a linear relation between stiffness and load, well established for Gaussian surfaces, is not obtained in this case. Instead, a power law, which can be motivated by dimensionality analysis, is a better descriptor. Also unlike Gaussian surfaces, we find that the stiffness curve is no longer independent of the Hurst exponent in this case. We carefully asses the possible convergence errors to ensure that our conclusions are not affected by them.

## Introduction

In the field of contact mechanics, the stiffness, i.e., the derivative of the load-separation curve, is one of the most relevant functional parameters used to characterize the contact between two rough bodies. Besides the relevance of stiffness on its own, it has also been directly linked to the contact thermal or electrical conductance^1^. Given its importance, it has been studied extensively in various contexts. Unexpectedly, the most thorough studies concern the normal contact of elastic bodies with friction- and adhesion-free interfaces^2–9^. In these studies, the contact of bodies with self-affine Gaussian surfaces against rigid flat bodies (which can be made equivalent to the contact of two bodies with rough surfaces^10^) has been fully characterized both numerically and theoretically. To favour repeatability, computer generated surfaces tend to be used instead of measured ones, as they provide for controllable, fault-free cases. By Gaussian surfaces it is here meant that their heights follow a Gaussian probability distribution. The self-affinity is given by their power spectrum which, for isotropic surfaces, is given as

1 $$\begin{aligned} C(q) = \left\{ \begin{array}{ll} C_0\left( \frac{\sqrt{q_x^{2}+q_y^{2}}}{q_0}\right) ^{-2\left( 1+H\right) } &{}\quad \text{ if }\, q_0 \le q \le q_1;\\ 0 &{}\quad \text{ otherwise }.\end{array} \right. \end{aligned}$$

where $q=\sqrt{q_x^2 + q_y^2}$ is the modulus of the wave-number, $C_0$ is a constant that determines the rms height of the surface, $q_1$ and $q_0$ are lower and upper wave-numbers and *H* is the Hurst exponent, which characterizes the decay of the power spectrum and can be related to the fractal dimension of the surface through $D_f = 3-H$. For clarity, it is easier to define wave-numbers in terms of cut-off wavelength as $q_i=2\pi /\lambda _i$. In this manner, $\lambda _0$ ($\lambda _1$) represents the longest (shortest) wavelength present in the surface. In a real nominally flat surface, $\lambda _0$ may be much smaller than the size of the surface and $\lambda _1$ goes down to the atomic size. When performing numerical studies, however, one is often constrained in the values one may pick due to computational limitations and must thus compromise and try to reduce convergence errors as much as possible. Once these values are fixed and the height lateral dimension are appropriately normalized (see Methods), *H* is the only parameter needed to characterize these surfaces. This is certainly a reason why the study of self-affine Gaussian surfaces is so appealing to researchers. Moreover, there have for long existed several readily available and easy to implement algorithms to generate these surfaces, see e.g.^5,11–13^. The simplicity to generate and characterize Gaussian, self-affine surfaces, together with the fact that they are an adequate representation for many natural and engineering surfaces^2^, has make their study very popular, leading to a well established characterization of their stiffness.

Without diminishing the relevance of the aforementioned self-affine Gaussian surfaces, some studies have pointed out that there are also other relevant applications in which non-Gaussian surfaces (i.e., surfaces with heights which do not follow a Gaussian distribution) are present and that the contact mechanics behaviour of these can differ significantly from that of Gaussian surfaces^14–19^. Despite that, very few studies have been dedicated to characterize the stiffness of such surfaces^19^ and it is thus much less understood. The lesser amount of studies on this area can probably be attributed to two difficulties one has traditionally encountered. The first one is that an efficient method to generate this type of surface with the desired degree of control (i.e., specifying well defined power spectrum and height distribution at the same time) has been unavailable^20^. To overcome this difficulty, a recent method presented by the authors can be used^21^. As outlined in the Methods, this allows full control of the power-spectrum and the height distribution. Therefore, it is possible to generate, and therefore study, well-controlled surfaces with arbitrary non-Gaussian height distributions. The second difficulty is that the characterization of this type of surfaces is far more complex than the Gaussian ones, as the label ’non-Gaussian’ encompasses an infinite number of very different distributions. To cope with this added complexity, most authors have used skewness and kurtosis to characterize the non-Gaussian height distribution^16,22,23^, which is not sufficient to fully specify (and thus control) them. As a result, the influence of a single parameter cannot be isolated. This difficulty is, unfortunately, unavoidable. Therefore, we will not attempt to study non-Gaussian surfaces in general but we will focus only on surfaces with heights following a Weibull probability distribution.

**Figure 1:**
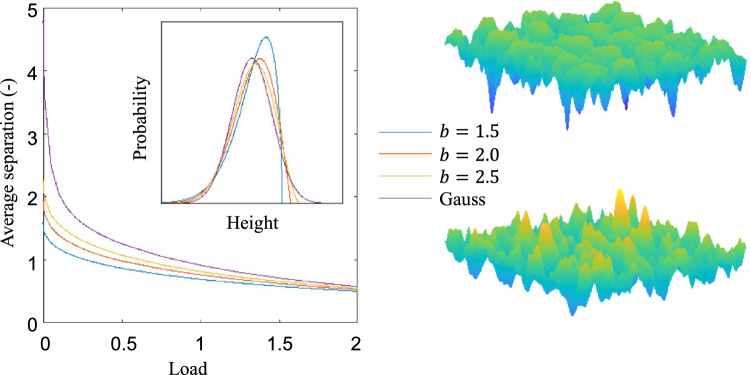
Left: Separation vs. contact pressure for a Gaussian surface and three Weibull surfaces with $b=1.5$, $b=2$ and $b=2.5$. On the insert, the corresponding height distributions. Right: Examples of surface realizations corresponding to a Gaussian surface (bottom) and a Weibull surface with $b=1.5$ (top).

For the purpose of this work, the Weibull distribution is defined as

2 $$\begin{aligned} f_W(z) = \left\{ \begin{array}{ll} \frac{b}{-(z-z_0)}\left( \frac{-(z-z_0)}{a}\right) ^{b-1}e^{-\left( -(z-z0)/a\right) ^b} &{}\quad \text{ if }\, (z-z_0)\le 0;\\ 0 &{}\quad \text{ otherwise }.\end{array} \right. \end{aligned}$$

where $b>0$ is the shape parameter, $a>0$ is the scale parameter, which can be fixed by setting a unitary value for the rms heights, and $z_0$ is the location parameter, which can be fixed by setting a zero mean of the heights. Note that the function is reversed in terms of $(z-z_0)$ with respect to the usual formulation. With this restrictions, the distribution introduces only one new parameter, *b*, which leads to a manageable study. The shape of this distribution is shown for three chosen parameters in Fig. 1 (insert), together with the Gaussian distribution. The relevance of this distribution is that it can be used to model surfaces that have suffered mild wear or plastic deformation, be it during operation or manufacturing. Indeed, a sufficiently low value of *b* suppresses the probability of the occurrence of high values of height (in fact, it is zero above a certain value). This results in the formation of a plateau with much shallower slopes and less prominent summits on the upper part of the surface, which would be expected in the aforementioned situations. Note that this approach gives a much more realistically looking surface than simply removing all the material above a certain threshold, as it does not create an unrealistic, totally flat plateau. Instead, the plateau formed, although capable of bearing a large load, is still rough. For the range of *b* studied here, an increase of *b* (e.g., between $b=2$ and $b=2.5$), leads to a distribution similar in nature but closer to the Gaussian one and thus the behaviour of the resulting surface can be expected to be closer to that of a Gaussian surface. Note, however, that it is not true that the Weibull distribution tends to a Gaussian one as $b\rightarrow \infty$. Instead, this distribution has a skewness of 0 and a kurtosis close to 3 (i.e., it is close to a Gaussian distribution) when $b\approx 3.6$ but then continues towards a negative skewness and a kurtosis larger than 3^24^.

The effect of *b* in the range considered is depicted in Fig. 1 (left). As expected, having flatter tops (smaller *b* for $b<3.6$) reduces the average separation at infinitesimally small loads (i.e., when the surfaces barely touch) thus making the response much stiffer at low loads. At higher loads, however, the similarity of the valleys make the response converge towards the Gaussian surface for all values of *b*.

Besides the previously given trivial description, a detailed analysis of the stiffness of surfaces with heights following a Weibull probability distribution has not been presented in the literature. Therefore, in this work we attempt to characterize in more detail the stiffness of these surfaces and how it evolves with increasing load. Moreover, we aim at comparing the stiffness of these surfaces with that of Gaussian surfaces, which has been studied in much greater detail. By that, we also expect to uncover which behaviours are common to all rough surfaces and which are specific to those with Gaussian height distribution.

## Results and discussion

The characterization of stiffness of self-affine fractal surfaces is not without difficultly, as the convergence with respect to the numerical parameters characterizing the fractal surface is rather slow. Following earlier works^25–27^, three non-dimensional parameters must be considered, i.e., $L/\lambda _0$, $\lambda _0/\lambda _1$ and $\lambda _1/\Delta x$, all of which must go to infinity in order to obtain a well resolved representative self-affine Gaussian surface. The first parameter relates the longer wavelengths to the size of the studied domain. If it is too small, the random fluctuations due to the stochastic nature of surface roughness cannot average out and the surface considered is not representative of all surfaces nominally equal. The second parameter, $\lambda _0/\lambda _1$, specifies the breath of the self-affine region. It it is too small, the self-similarity of the surface is lost. Finally, $\lambda _1/\Delta x$ compares the grid size, $\Delta x$, with the smallest wavelengths present in the roughness, $\lambda _1$. The ratio must be small enough for these to be well resolved. Clearly, increasing all three parameters at the same time is computationally challenging, since $\lambda _0$ and $\lambda _1$ can be found both at their numerator and denominator. Moreover, insufficiently large values can lead to spurious effects, as described below. Therefore, before studying the surfaces with Weibull height distributions, the stage is set by reviewing the stiffness of Gaussian surfaces and characterizing the effect of not having sufficiently large values on them.

### Gaussian surfaces

**Figure 2:**
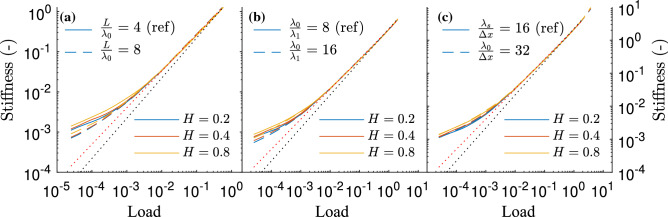
Effect of varying the different fractal non-dimensional parameters on the stiffness curve of a Gaussian surface; $L/\lambda _0$ (**a**), $\lambda _0/\lambda _1$ (**b**) and $\lambda _1/\Delta x$ (**c**). In all cases the reference case is characterized by $L/\lambda _0 = 4$, $\lambda _0/\lambda _1= 8$ and $\lambda _1/\Delta x = 16$ and only the specified parameter is changed. The black dotted line indicates a linear relation while the red one marks a power law with exponent 0.9.

The stiffness of self-affine surfaces with Gaussian height distribution has been theoretically predicted to increase linearly with load, in a fashion independent of the Hurst exponent^4^. As expressed by Müser and Wang^28^, this result derives from the observation that, when load increases, one finds “more of the same”, i.e., that the contact stress distribution and the size distribution of the contact patches remain unchanged, and the contact only evolves by adding more contact patches. As also argued by Müser and Wang^28^, however, this cannot possibly hold exactly, as the larger contact patches are severely suppressed at low loads. Moreover, when the contact is very small (following Müser and Wang^28^, the threshold can be roughly estimated to be at a value of $\pi (2-H)\lambda _s^2/(16(1-H))$), the patches behave independently instead of as contacts pertaining to a self-affine surface. Therefore, the Hertzian theory^29^ would be more suitable to study them. These limits, however, leave a sufficiently broad range of contact sizes in which this “more-of-the-same” assumption is a good approximation and a linear relation is usually found between stiffness and load^3,6^. The need for the aforementioned assumption is highlighted when studying cases where the linear relation is not observed. For example, Pohrt and Popov^7^ observed that, as load decreased, the linear relation turned into a power law relation with an exponent smaller than one, dependent on the Hurst exponent. This was later related by Pastewka et al.^6^ to a size effect, i.e., a small value of $L/\lambda _0$. In particular, they could show that the transition from the linear to the power law relation occurred at ever lower loads with increasing $L/\lambda _0$. One can therefore infer that, on the limit $L/\lambda _0\rightarrow \infty$, the linear relation should hold for all loads. A theoretical discussion supporting this reasoning was also presented by Pastewka et al.^6^. One can also attain an intuitive explanation for the failure of the linear relation at low loads and why it is enhanced for small domains by connecting it with the number of contact patches. When the load is sufficiently small for the linear relation not to hold (the reader is referred to the discussion around Fig. 2 for a numerical estimate), the waves with wavelength close to $\lambda _0$, which typically have an amplitude larger than waves with smaller wavelengths, will dominate the location of the initial contact points. Therefore, the initial contact spots will appear separated by a distance of the order of $\lambda _0$. If $L/\lambda _0$ is small, very few of this contacts will be present. With so few contacts, the “more-of-the-same” assumption discussed by Müser and Wang^28^ cannot hold and the linear relation is lost. As load increases, however, more and more contact will appear and these will start to interact with each other. Eventually, there is enough contacts of various sizes so that the “more-of-the-same” assumption holds and the linear relation is recovered. The linear relation can also be recovered by increasing the ratio $L/\lambda _0$. Indeed, as this ration increases, more contact patches are present at the initial stage of the loading and the linear relation can be recovered at a lower load. One can also note that, following a similar reasoning, one a too low breath of the fractal region, $\lambda _0/\lambda _1$ should also be expected to cause the linear relation to disappear. Finally, having a too low resolution ($\lambda _1/\Delta x$) can be expected to lead to errors in the computation of the stiffness, which should be expected to be larger at smaller loads. It is thus clear that a good characterization of the stiffness requires sufficiently large values for the three parameters characterizing the numerical representation of the self-affine surface.

In Fig. 2, the stiffness of self-affine surfaces with Gaussian height distribution for various values of *H*, $L/\lambda _0$, $\lambda _0/\lambda _1$ and $\lambda _1/\Delta x$ is depicted. A reference case (depicted as a solid line in the three sub-figures) is first considered. This case is given by $L/\lambda _0 = 4$, $\lambda _0/\lambda _1= 8$ and $\lambda _1/\Delta x = 16$, which leads to a grid-size of $512\times 512$ points, and three values of the Hurst exponent are considered. The stiffness of this reference case behaves in a manner similar to what should be expected according to the previous discussion. At sufficiently large loads, the stiffness is close to a linear relation independent of *H*, while at low loads a power law with exponent smaller than one appears. It is apparent, however, that the curve deviates slightly from the linear trend even at large loads. Indeed, the best fit to a power law obtained when considering load larger than $5\times 10^{-3}$, depicted in red in Fig. 2, gives an exponent of 0.90 instead of 1. This value is, nevertheless, (almost) independent of *H*. The exponent depends, however, on the range of loads used, as it increases if only larger loads are considered. For instance, it becomes 0.95 if one only considers loads larger than $5\times 10^{-2}$ ($\approx 0.5$% real contact area) and 0.99 if the cut-off is set at a load of $5\times 10^{-1}$ ($\approx 5$% real contact area). As a final comment, one can observe that, as expected from the discussion above, the stiffness at low loads does depend on the Hurst exponent.

Let us now focus of the effect of increasing domain size, i.e., increasing $L/\lambda _0$ while keeping everything else unchanged. In Fig. 2a it is apparent that the stiffness approaches the linear relation when $L/\lambda _0$ is increased. This is particularly noticeable at low loads, since the departure from the power law occurs at a lower load. This occurs, indeed, as discussed in the opening of this section. In some results available in the literature (see, e.g., Fig. 1 by Pastewka et al.^6^), an almost perfect linear relation is observed until the curves with different $L/\lambda _0$ deviate from each other. This behaviour, which follows the expectations given by the discussion above, is not observed here. Instead, the curves split at a load of $5\times 10^{-3}$ and the trend considering loads larger than this value is best fitted by a power law with exponent 0.90. An explanation for this discrepancy between the results presented here and those by Pastewka et al.^6^ is probably related with the breath of the fractal spectrum, given by $\lambda _0/\lambda _1$. As seen in Fig. 2b, increasing this value also leads to the curves approaching the linear relation. Moreover, this also explains why the exponent of the fitted power law tends to 1 as load increases. At higher loads, the longer wavelengths dominate the stiffness by being flattened and adding smaller wavelengths do not change it substantially. At lower loads, however, the longer wavelengths cannot be flattened as severely and thus the detail of the contact at their tops become more relevant. Therefore, one can anticipate that the expected behavior, similar to that presented by Pastewka et al.^6^, should be recovered as $\lambda _0/\lambda _1$ increases. It should be noticed, however, that the convergence towards a linear relation between stiffness and load as $L/\lambda _0\rightarrow \infty$ and $\lambda _0/\lambda _1\rightarrow \infty$ has been shown to be rather slow^3,9^. In fact, $\lambda _0/\lambda _1=512$ was used by Pastewka et al.^6^ to obtain their results. Incidentally, a power law similar to the one presented here has been found elsewhere in the literature (see e.g. Paggi and Barber^5^) using a similar range of parameters controlling the numerical representation of the surface. Looking finally at the effect of $\lambda _1/\Delta x$, a much smaller effect can be observed in Fig. 2c, which indicates that the reference value of $\lambda _1/\Delta x=16$ was already sufficient and maybe even larger than necessary.

### Weibull surfaces

**Figure 3:**
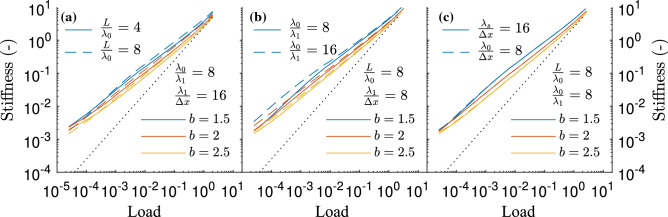
Effect of varying the different fractal non-dimensional parameters on the stiffness curve of a surface with Weibull height distribution; $L/\lambda _0$ (**a**), $\lambda _0/\lambda _1$ (**b**) and $\lambda _1/\Delta x$ (**c**). The value of the parameters not varied are indicated in each sub-figure. The black dotted line indicates a linear relation.

Having reviewed the convergence of Gaussian surface, the attention is now directed to the Weibull surfaces. The convergence of their stiffness with $L/\lambda _0$, $\lambda _0/\lambda _1$ and $\lambda _1/\Delta x$ is depicted in Fig. 3 for $H=0.8$ and three values of *b*. Placing first the attention to $L/\lambda _0$ (Fig. 3a), the behaviour is not as expected given the results for Gaussian surfaces. Unlike in that case, the major differences with increasing $L/\lambda _0$ do not occur solely at low loads but are rather spread throughout the whole load range studied. This could hint at an intrinsic difference between Weibull and Gaussian surfaces. However, it is more likely related to a shortcoming of the method to generate fractal surfaces with non-Gaussian distribution which, as discussed in Methods, might introduce errors in surfaces with a too low value of $L/\lambda _0$. At a value of $L/\lambda _0=8$, however, the surface can be expected to be generated with sufficient accuracy. The study is therefore followed with $L/\lambda _0=8$. Given the results in the previous section and in order to restrict the grid size while keeping $L/\lambda _0=8$, the parameter $\lambda _1/\Delta x$ is considered. The convergence of the stiffness curve with this parameter is depicted in Fig. 3c. It is clear that halving its value to $\lambda _1/\Delta x=8$ with respect to the reference value does not affect significantly the results. Keeping this value of $\lambda _1/\Delta x$, and still having $L/\lambda _0=8$, the fractal breath, $\lambda _0/\lambda _1$ is finally considered. In this case, the expectations are met and the errors are concentrated at low loads. At high loads, the low wavelengths have a smaller impact and thus their absence is felt to a lesser extent. Importantly for the comparison made in the following section, the stiffness deviates further away from the linear relation. Moreover, the effect of the breath is smaller than that of the domain size at higher loads. Because of this, it seems reasonable to choose the case characterized by $L/\lambda _0=8$, $\lambda _0/\lambda _1=8$ and $\lambda _1/\Delta x=16$ to compare these surfaces with the Gaussian ones.

### Comparison between Gaussian and Weibull surfaces

**Figure 4:**
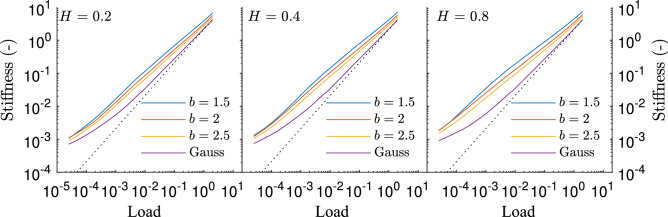
The stiffness of surfaces with Gaussian or Weibull height distributions, for three values of the Hurst exponent *H*. The black dotted line indicates a linear relation.

Based on previous discussion, $L/\lambda _0=8$, $\lambda _0/\lambda _1=8$ and $\lambda _1/\Delta x=16$ are chosen to compare the surfaces with Gaussian height distribution with those with Weibull height distribution. Their stiffness, for three different values of the Hurst exponent, is depicted in Fig. 4.

If one first looks at the lower loads, the behaviour of the Weibull surfaces seem to be completely different from that of the Gaussian surfaces. Given the convergence errors shown in Fig. 3b, however, it would be risky to venture describing these differences here. It is clear, however, that much broader fractal breath is needed to obtain a consistent result as compared to what is needed for Gaussian surfaces. Possibly, this need could be related to the flatness of the tops. Indeed, by decreasing the breath, the tops might become artificially too flat. Therefore, one might conjecture that, with a larger breath, these tops turn rougher and the curves tend towards a behaviour similar to that of Gaussian surfaces, as it might be insinuated by the case $H=0.8$.

**Figure 5:**
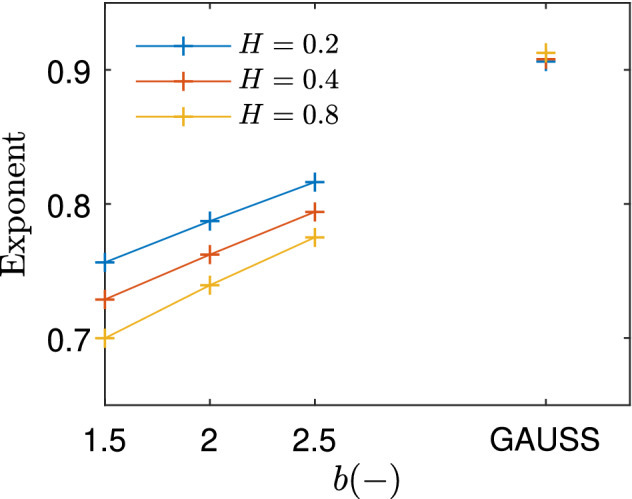
Best fit exponent for the curves in Fig. 4, considering loads above $5\times 10^{-2}$. The results corresponding to the Gaussian surfaces are placed at $b=3.6$, since at this value the Weibull distribution is at its closest to the Gaussian one.

At sufficiently large loads, i.e., around $p>5\times 10^ {-2}$, the stiffness can be described by a power law with respect to the contact pressure. Unlike the case of Gaussian surfaces, where the exponent tends to one, the exponent of Weibull surfaces decreases with decreasing *b*, as depicted in Fig. 5. This trend can be related to the shape of the surface tops. The smaller *b* is, the flatter these tops are. Since a flatter top is also stiffer, the overall stiffness of the surface decreases to a lesser extent when load is reduced. It is also apparent that the differences between different Hurst exponents become more acute as *b* decreases. Moreover, a linear relation seems to hold between the exponent and *b* for a given Hurst exponent. Noting that the Weibull distribution becomes rather close to a Gaussian one when $b\approx 3.6$, the obtained exponents for the Gaussian distribution are placed at this point in Fig. 5. Extending the lines corresponding to the Weibull cases, it seems apparent that the linear relation can be extended towards values of *b* larger than what has been studied.

## Discussion

The results presented here are consistent with the linear relation already indicated in the literature for the case of surfaces with Gaussian height distribution^3,4,6^. One should bear in mind, however, that convergence with increasing fractal parameters, specially $L/\lambda _0$ and $\lambda _0/\lambda _1$ is notoriously difficult to achieve. Indeed, the deviation from the linear relation in several works in the literature^5,7,9^, as well as the ones presented here, can be linked to insufficient values of $L/\lambda _0$ and/or $\lambda _0/\lambda _1$. This result can probably be generalized by noting that, at the loads studied, only the higher peaks engage in contact. Therefore, it can be hypothesized that any surface with a height distribution with an upper tail similar to a Gaussian distribution will behave similarly as a Gaussian one. An example of such surfaces could be a surface with a Weibull height distribution reversed in comparison to the ones studied here. The height distribution of the surfaces considered here, however, have an upper tail significantly different than a Gaussian distribution and thus their higher peaks look completely different. Because of that, the stiffness is quite different from that observed in Gaussian surfaces, to the point where the linear relation is no longer observed. Instead a power law relation, with exponents within the range 0.7–0.8 seem to fit the observations.

To make sense of the observed difference, it is interesting to recall the derivation presented by Paggi and Barber^5^. They established a power law relation between stiffness and load based on arguments of dimensional analysis and incomplete self-similarity. This result was shown to be consistent with other (more specific) theoretical ones. This, of course, also includes the linear relation valid for Gaussian surfaces^4^, which is simply a power law with exponent 1. Based on the results presented here, it is apparent that a power law relation should be used to describe the stiffness curve of a general surface. In the particular case of a Gaussian surface, the exponent becomes one and thus a linear relation is observed (provided that the values of $L/\lambda _0$ and $\lambda _0/\lambda _1$ are sufficiently large). In other cases, such as the Weibull surfaces studied here, however, it seems that the exponent can differ from unity even when convergence issues are addressed. One can therefore conclude that a power law is valid for a fairly general type of surface, with the linear relation being an important specific case.

## Methods

### Computation of stiffness

The boundary element based model for friction-less elastic contact presented by Sahlin et al.^30^ is used in this work to solve the contact mechanics problem needed to compute the separation at a given load. This model reduces the dimensionality of the problem so that only a two-dimensional (rectangular) grid is used to solve for the three-dimensional problem. To achieve this, Love’s solution^31^ for the deformation caused at the surface by a uniform pressure distribution over a rectangular element is used. Then, the DC-FFT approach^32^, which uses fast Fourier transform to accelerate the computation, is used to efficiently compute the deformation caused by a given pressure distribution. Then, the pressure is solved for by minimizing the complementary potential energy. At each relaxation step, the load is balanced by shifting the pressure distribution upwards or downwards, until a relative tolerance of $10^{-3}$ is reached with respect to the target value. The relaxation is terminated when all points in contact, i.e., those points under positive pressure, are in the contact plane. The tolerance used here is $10^{-6}h_{rms}$, where $h_{rms}$ is the rms-height of the surface roughness. In order to compute the stiffness, the separation is computed at a number of target loads and at loads 1% larger. The stiffness, which is the derivative of the separation-load curve, is then estimated using first order finite differences between these two values of pressure. The results are computed and presented in a non-dimensional form. For this, the following reference parameters for pressure, $p_r$, height, $h_r$, and lateral dimension, $x_r$, are used,

3 $$\begin{aligned} p_r = \frac{\pi E^*h_{rms}}{L}, \quad x_r = L,\quad h_r = h_{rms}, \end{aligned}$$

where *L* is the length of the contact and $E^*=E/(1-\nu ^2)$, with *E* and $\nu$ being the elastic modulus and Poisson ratio.

### Fractal surface generation

To generate self-affine fractal surfaces with Gaussian height distribution, the classical method presented by Hu and Tonder^11^ is used. In order to generate the surfaces with a Weibull height distribution, a method newly proposed by the authors have been employed^21^. The method to generate Gaussian surfaces is the simplest of the two. Following Hu and Tonder^11^, one starts with white noise with a Gaussian height distribution, which can be obtained by sampling uncorrelated values from a Gaussian distribution. Then, one applies a filter in the Fourier space so that the desired power spectrum (Eq. 1) is obtained. Since the Gaussian distribution is preserved under such filter, the resulting surfaces have the desired power spectrum and a Gaussian height distribution. When a sample with a non-Gaussian height distribution is filtered in this manner, however, its height distribution is not preserved. To allow for keeping the specified height distribution, Pérez-Ràfols and Almqvist^21^ implemented a two-step method. Starting from a sample of white noise with the desired height distribution, the two steps applied iteratively are (i) a filter is used in the Fourier space to obtain the correct power spectrum, and (ii) the height distribution is corrected via rank ordering. Under right conditions, discussed in detail by Pérez-Ràfols and Almqvist^21^, this method readily converges and a surface is obtained with the desired power spectrum and height distribution.

A limitation relevant to this work is that the resulting surface might not be accurate if the value of $L/\lambda _0$ is too small. The cause of this error is that the height distribution is achieved too exactly. To understand why this is a problem, one may first look at the generation of Gaussian surfaces with a small value of $L/\lambda _0$. In this case, the height distribution obtained for a given realization should be expected to deviate quite significantly form the desired Gaussian distribution, see e.g. Yastrebov et al.^33^. This is because the surface is too small for a good average to be obtained. Only when several realizations are considered together do the heights approach closely to the Gaussian distribution. This behaviour should also be expected when the surface is characterized by any non-Gaussian height distribution. With the algorithm used here, however, the heights are forced to conform to the given distribution much better than they would otherwise. Indeed, the heights can be made to follow closely the desired distribution even for $L/\lambda _0=1$. This changes the nature of the surface in ways that have not yet been studied. For sufficiently large values of $L/\lambda _0$, however, the aforementioned issue does not pose any problem. A value of $L/\lambda_0=8$ should be sufficient to obtain trust-worthy results^21^.
